# DNA-PKcs restricts *Zika virus* spreading and is required for effective antiviral response

**DOI:** 10.3389/fimmu.2022.1042463

**Published:** 2022-10-13

**Authors:** Daniel de Oliveira Patricio, Greicy Brisa Malaquias Dias, Lucilene Wildner Granella, Ben Trigg, Helena Claire Teague, Dina Bittencourt, André Báfica, Alfeu Zanotto-Filho, Brian Ferguson, Daniel Santos Mansur

**Affiliations:** ^1^ Laboratório de Imunobiologia, Departamento de Microbiologia, Imunologia e Parasitologia, Universidade Federal de Santa Catarina, Florianópolis, Brazil; ^2^ Department of Pathology, University of Cambridge, Cambridge, United Kingdom; ^3^ Laboratório de Farmacologia e Bioquímica do Câncer, Departamento de Farmacologia, Universidade Federal de Santa Catarina, Florianópolis, Brazil

**Keywords:** *Zika virus*, interferon, DNA-PKcs, double-strand DNA breaks, infection

## Abstract

*Zika virus* (ZIKV) is a single-strand RNA mosquito-borne flavivirus with significant public health impact. ZIKV infection induces double-strand DNA breaks (DSBs) in human neural progenitor cells that may contribute to severe neuronal manifestations in newborns. The DNA-PK complex plays a critical role in repairing DSBs and in the innate immune response to infection. It is unknown, however, whether DNA-PK regulates ZIKV infection. Here we investigated the role of DNA-PKcs, the catalytic subunit of DNA-PK, during ZIKV infection. We demonstrate that DNA-PKcs restricts the spread of ZIKV infection in human epithelial cells. Increased ZIKV replication and spread in DNA-PKcs deficient cells is related to a notable decrease in transcription of type I and III interferons as well as *IFIT1*, *IFIT2*, and *IL6*. This was shown to be independent of IRF1, IRF3, or p65, canonical transcription factors necessary for activation of both type I and III interferon promoters. The mechanism of DNA-PKcs to restrict ZIKV infection is independent of DSB. Thus, these data suggest a non-canonical role for DNA-PK during *Zika virus* infection, acting downstream of IFNs transcription factors for an efficient antiviral immune response.

## Introduction

*Zika virus* (ZIKV) is a single-strand RNA mosquito-borne flavivirus (1). First isolated in 1947 in Africa, ZIKV caught public health attention in 2007 with the first viral outbreak in Pacific Islands, from where it spread to South America in 2015 (2–5). In 2016, Zika disease was declared to be a worldwide public health emergency due to severe neurological manifestations in newborns (6, 7). The neurological complications are associated with the tropism of ZIKV for human neural progenitor cells which results in growth arrest, DNA double-strand breaks (DSBs), and cell death (8, 9). DSBs are the most cytotoxic type of DNA lesions that rapidly activate DNA-damage repair response, orchestrated in part by the DNA-dependent protein kinase (DNA-PK) complex (10, 11). However, the relevance of DNA-PK in restricting ZIKV infection is unknown.

DNA-PK is a multifunctional protein complex consisting of Ku70, Ku80, and the catalytic subunit (DNA-PKcs) which are involved, among other functions, in DNA damage repair, V(D)J recombination of lymphocytes receptors, transcriptional regulation, DNA replication, and RNA metabolism (11–13). Present in the cytoplasm and nuclei, DNA-PK also functions as an intracellular DNA receptor critical for primary immune response against DNA virus infections by inducing interferon (IFN)-I and IFN-III expression (14–19). IFNs induce the transcription of interferon-stimulated genes (ISGs) that are critical for inhibition of viral replication cycle (20–22).

During RNA virus infections, IFN-I and IFN-III are mainly induced by intracellular RNA sensing receptors such as retinoic acid-inducible gene I (RIG-I) and melanoma differentiation-associated protein 5 (MDA5) (23). For instance, ZIKV RNA genome detection is mediated by RIG-I, leading to activation and nuclear translocation of the transcription factors belonging to the interferon regulatory factor (IRF) and nuclear factor-kappa B (NF-κB) families (24–35). IRFs and NF-κB bind to interferon-stimulated response element (ISRE) and NF-κB motifs, respectively, both present in the promoter region responsible for IFN-I and IFN-III genes (32, 36).

Several studies associate DNA-PK complex with ZIKV or other flaviviruses, such as *Dengue virus* (DENV). Vetter et al. (37) described DNA-PK localization and activation as a very early marker of DENV infection (37). DENV infection causes DNA-PK subunits relocation to the nucleoli, which may regulate RNA splicing (37–39). In addition, human cells with Ku80 protein partially depleted, reduce the interferon response induced by DENV (37). The DNA-PK complex is also associated with both ZIKV and DENV genomic RNA in human cells, with unknown effects (40). In this context and considering ZIKV can induce DSB (8, 9), we investigated whether DNA-PKcs affects ZIKV infection and triggers antiviral immune response pathways.

We found that DNA-PKcs restricts ZIKV spread in human epithelial cells. In the course of infection, DNA-PKcs is required for IFN-related gene transcription, independent of transcription factors IRF1, IRF3, or p65 (NF-κB subunit). In addition, DNA-PKcs role during ZIKV infection was DSB independent. This study provides information about the DNA-PKcs dynamics on the antiviral immune response during ZIKV infection and may contribute to new therapeutic strategies.

## Materials and methods

### Cell culture and reagents

A549 (ATCC^®^, CCL-185™), RPE (ATCC, CRL-2302), Vero (ATCC^®^, CCL-81™), and derived cell lines were maintained in DMEM-F12 (GIBCO, 12400-024) containing 5% fetal bovine serum (FBS – GIBCO, 12657-029) supplemented with 1 U/mL penicillin/streptomycin (Sigma, P4333). C6/36 (ATCC^®^, CRL-1660™) cells were maintained at 28 °C, in L-15 (Sigma, L4386) containing 5% FBS supplemented with 0.26% tryptose phosphate broth (GIBCO, 18050-39), and 1 U/mL penicillin/streptomycin. Both cell lines were routinely tested for mycoplasma contamination. Cells were treated with 0.5 or 1 µM of NU7441 (BioGems, 5039598), 100 ng/mL human TNF (Peprotech, 300-01A), and 3 µM etoposide (Sigma, E1383).

### Virus infection

ZIKV strain BR2015/15261 (41) was kindly provided by Dra. Claudia N. Duarte dos Santos (Fiocruz-PR, Brazil). Viral stocks were purified from infected C6/36 cells supernatants and titrated by plaque assay on Vero cells. This virus was used for infection for indicated times and multiplicity of infection (m.o.i.).

### Flow cytometry

Cells were detached from the plate with trypsin-EDTA (GIBCO, 25300-062), centrifuged at 460 x *g* for 5 min, washed in saline solution, and stained with Zombie NIR™ fixable viability kit (Biolegend, 423105) at the dilution 1:2000 for 20 min at room temperature. Cells were then fixed with 3% paraformaldehyde (PFA – Sigma, P6148) for 20 min followed by staining with FITC-conjugated flavivirus E protein antibody (anti-4G2) (provided from Fiocruz-PR, Brazil) in permeabilization buffer (0.25% saponin - Vetec, 1364) for 40 min. All cells were washed with saline solution and acquired on BD FACSVerse with FACSuite software. Analysis was performed using FlowJo software v. 10.1 (TreeStar). The gating strategy used for flow cytometry is available in **Supplementary Figure 2**.

### MTT assay

Cells were incubated with 0.5 mg/mL MTT reagent (Amresco, 793) for 3 hours at 37 °C and 5% CO_2_, followed by incubation of DMSO for formazan crystals extraction. Measures were performed at 570 nm abs on a Biotek spectrophotometer using Gen5 1.10 software.

### Immunofluorescence

Cells were seeded onto a plate containing a 15 mm glass coverslip, followed by ZIKV infection at determining time and concentration. Cells were then fixed with 3% PFA for 20 min followed by permeabilization with PBS containing 0.1% Triton X-100 (Amresco, 694) and 2% bovine serum albumin (BSA – Inlab, 1870) for 5 min. Primary antibodies (**Supplementary Table I**) were diluted in PBS with 2% BSA, and incubated for 1 hour at room temperature, followed by detection by secondary antibodies (**Supplementary Table II**), diluted in PBS with 2% BSA, and incubated for 1 hour at room temperature. Then, cell nuclei were stained with DAPI (Molecular probes, D3571) for 15 minutes and mounted with Mowiol (Sigma, 81381). Pictures were obtained on an Olympus BX41 microscope and processed on Q-capture Pro 5.1 software (Q-imaging). For transcription factors translocation into the nucleus, cells were counted on at least 20 infection plaque from each sample, in biological triplicates using ImageJ software.

### Real-time quantitative PCR

Total RNA was extracted from cells using TRIzol reagent (Ambion, 15596026) according to manufacturer’s instructions. RT-PCR was performed with M-MLV Reverse Transcriptase (Promega, M170A) using 500 ng of RNA. For qPCR reaction, 2 µL of 1:20 diluted cDNA was used in a final volume of 10 µL, and 0.1 µM forward and reverse primers (**Supplementary Table III**), performed with GoTaq qPCR Master Mix (Promega, A600A). *GAPDH* was used as the housekeeping gene. Data were obtained on the StepOne Plus Real-Time PCR system (Applied BioSystems) and analyzed with StepOne Software v2.1. Relative mRNA expression was calculated by 2^-ΔΔCT^ method.

### Immunoblotting

Immunoblotting was used for characterization of A549 *^PRKDC-/-^* cells. Cells were lysed using a lysis buffer containing protease inhibitor (Mini Protease Inhibitor Tablets – Roche, 5056489001). The samples were incubated for 30 min at 4 °C, being briefly vortexed each 10 min followed by centrifugation at 13.000 x *g* for 10 min at 4 °C. The supernatant was transferred to a new tube and the total proteins were quantified using Pierce BCA protein assay kit (Thermo, 23225). From total proteins, 20 µg were transferred to polyacrylamide gel electrophoresis for protein separation and then transferred to nitrocellulose 0.22 µm blotting membranes. The membranes were blocked in 5% non-fat milk in TBS containing 0.1% Tween 20 (TBST) for 1 hour at room temperature. Membranes were then probed with primary antibodies (**Supplementary Table IV**) diluted in TBST containing 5% BSA, at 4 °C shaking overnight. Membranes were washed with TBST and incubated in secondary antibodies (**Supplementary Table V**) for 1 hour at room temperature. Then, membranes were washed and chemiluminescence developed using ECL substrate (Pierce, 34577). Tubulin was normalized as the reference control.

### CRISPR/Cas9

A549 *^PRKDC-/-^* and RPE *^PRKDC-/-^* cells were generated with a pair of sgRNA guides (Guide1: GATCACGCCGCCAGTCTCCA; Guide2: CAGACATCTGAACAACTTTA). The guides were inserted in pX458 plasmids containing Cas9 encoded genes plus GFP sequence for clone isolation. The cells were transfected with pX458/Guide using lipofectamine 3000 reagent (Invitrogen, L3000-008) following manufacturer’s instructions. For clone selection and expansion, the fluorescent cells were isolated into a 96-well plate using BDMelody cell sorter (BD). Knockout cell line clones were confirmed by immunofluorescence and immunoblotting assays.

### Data processing and statistical analyses

Data derived from the experiments were processed using GraphPad Prism nine software. The data were analyzed according to experimental settings using unpaired two-tailed Student’s *t*-test or two-way ANOVA, with Sidak’s correction where necessary.

## Results

### DNA-PKcs is required for control of ZIKV infection

**Figure 1:**
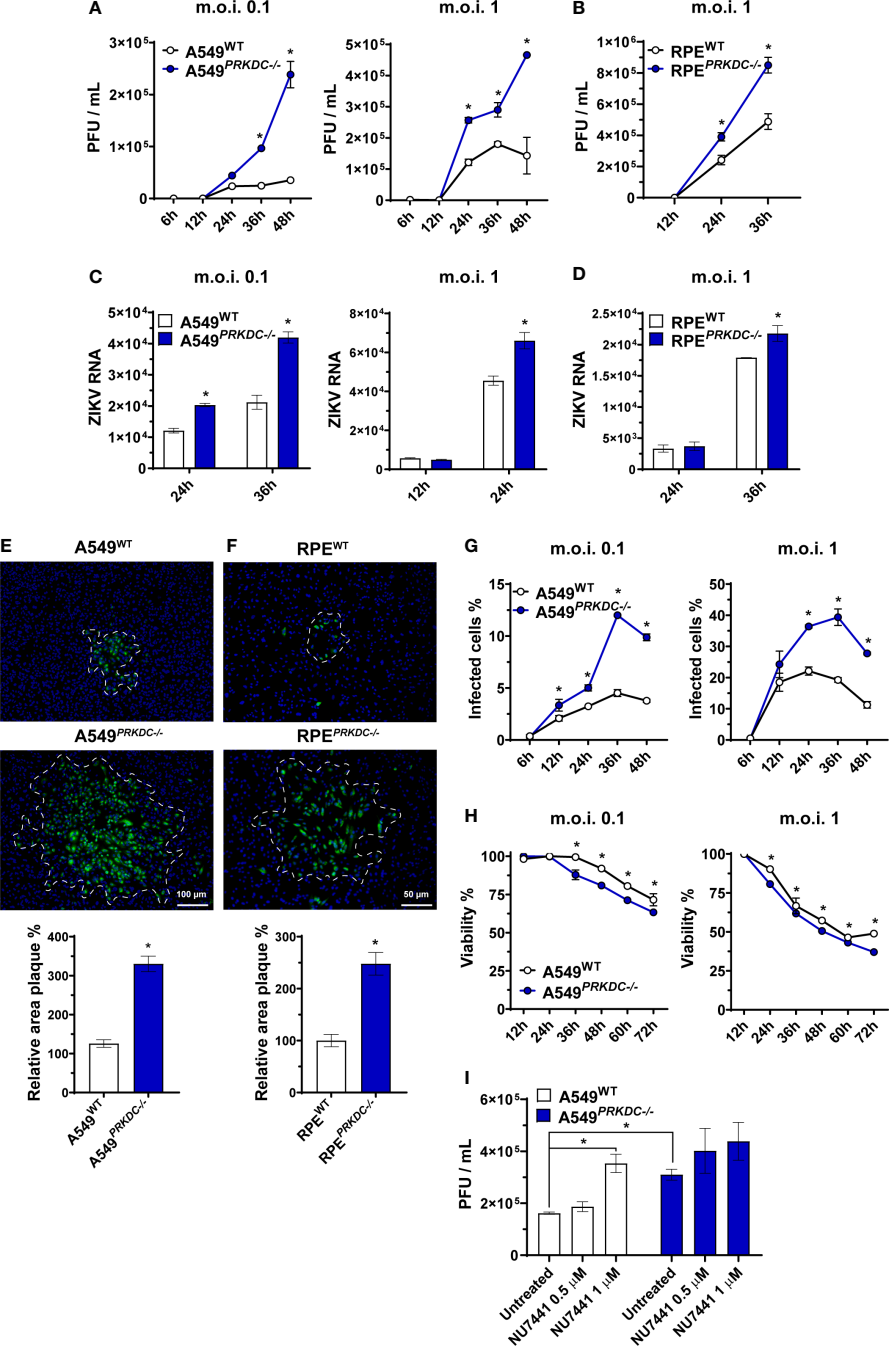
DNA-PKcs is critical for control of ZIKV infection. Virus replication measured by plaque assay, expressed as plaque-forming units per mL (PFU/mL), on **(A)** A549^WT^ and A549*^PRKDC-/-^* or **(B)** RPE^WT^ and RPE*^PRKDC-/-^* cells infected with ZIKV at indicated m.o.i. and time. RT-qPCR analysis to measure ZIKV RNA in **(C)** A549^WT^ and A549*^PRKDC-/-^* or **(D)** RPE^WT^ and RPE*^PRKDC-/-^* cells infected at indicated m.o.i. and time. **(E)** A549^WT^ and A549*^PRKDC-/-^* or **(F)** RPE^WT^ and RPE*^PRKDC-/-^* cells infected with 50 PFU of ZIKV at 48 hours in semi-solid medium, then ZIKV-E protein (green) was stained for immunofluorescence analysis, and the relative area of infection percentage was measured using the ImageJ software. The cell nuclei were stained with DAPI (blue). **(G)** Percentage of ZIKV-infected A549^WT^ and A549*^PRKDC-/-^* cells at indicated m.o.i. and time, analyzed by flow cytometry. **(H)** Viability analysis by MTT assay of ZIKV-infected A549^WT^ and A549*^PRKDC-/-^* cells relative to uninfected cells (mock) at indicated m.o.i. and time. **(I)** A549^WT^ and A549*^PRKDC-/-^* were pretreated with NU7441 (0.5 and 1 µM) at 24 hours followed by ZIKV infection (m.o.i. 1) at 24 hours. We used two-way ANOVA with Sidak’s correction in **(A–D, G, H)**, and unpaired two-tailed Student’s t-test was used in **(E, F)**. * p<0.05, n = 3, error bars ± SEM.

DNA-PK complex plays a critical role in DNA damage repair such as DSB as well as in antiviral immune response (10, 11, 14, 16–19). It was demonstrated that ZIKV can induce DSB in neural progenitor cells (8, 9). However, the role of the catalytic subunit of DNA-PK in controlling ZIKV infection is currently unknown. To determine the role of DNA-PKcs during ZIKV infection, we used CRISPR/Cas9 editing of the *PRKDC* gene to generate DNA-PKcs-deficient A549 and RPE epithelial cells, referred to as A549 *^PRKDC-/-^* (**Figures S1A, B**) and RPE *^PRKDC-/-^* (**Figure S1C**). We observed an increase of infectious ZIKV particles released from A549 *^PRKDC-/-^* (**Figure 1A**) and RPE *^PRKDC-/-^* (**Figure 1B**) compared with wild-type (WT) cells as well as a significant increase in intracellular ZIKV RNA (**Figures 1C, D**). In the absence of DNA-PKcs, ZIKV spread to adjacent cells, as measured by plaque area size (**Figures 1E, F**), as well as the percentage of infected cells (**Figure 1G**, **Figure S2**) and dead cells (**Figure 1H**) are also increased. Altogether, these results indicate DNA-PKcs is required for full control of ZIKV infection in both A549 and RPE cells.

To evaluate whether the DNA-PKcs kinase function is required for ZIKV control, we treated A549 cells with NU7441, a DNA-PKcs inhibitor (42, 43), 24 hours before infecting with ZIKV (**Figure 1I**). Similar to what we observed in *PRKDC-/-* cells, NU7441 pre-treatment increased ZIKV infection in WT, but not in A549 *^PRKDC-/-^* cells. These results suggest DNA-PKcs kinase function is necessary for control of ZIKV infection.

### ZIKV infection does not induce double-strand DNA breaks in A549 cells

**Figure 2:**
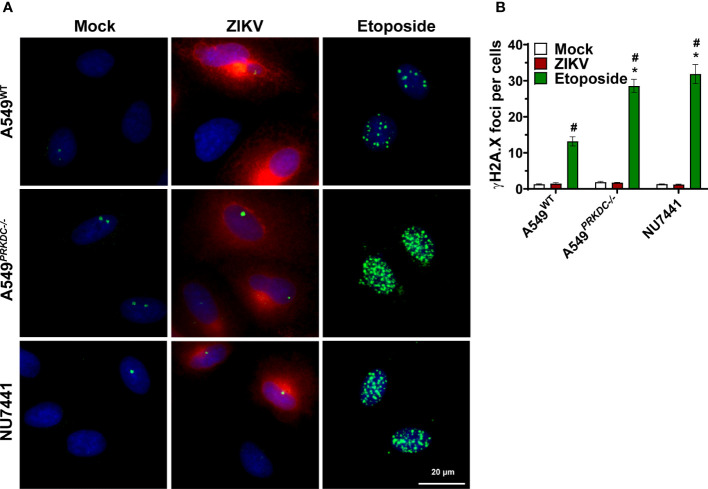
ZIKV infection does not induce DSB in A549 cells. A549^WT^, A549*^PRKDC-/-^* and 1 µM NU7441 pre-treated A549^WT^ infected with ZIKV (m.o.i. 1) at 24 hours. Stimulation with 3 µM etoposide for 12 hours was used as a DSB positive control. **(A)** Immunofluorescence to analyze γH2AX (green) in the ZIKV-infected cells (red, ZIKV-E protein). The cell nuclei were stained with DAPI (blue). **(B)** Percentage of γH2AX foci per cell showed in **(A)**. *Compared with WT cells; #Compared with mock. We used two-way ANOVA with Sidak’s correction. * or # p<0.05, n = 3, error bars ± SEM.

IFN-I is induced by DNA-PKcs through its DNA sensing function following DSB (44). Furthermore, ZIKV infection induces DSB in neural progenitor cells as observed by γH2A.X histone phosphorylation (8, 9). Thus, we evaluated whether ZIKV infection induces DSB in A549 cells, leading to a source of immunostimulatory DNA to activate DNA-PKcs. To assess this, the A549^WT^ and A549 *^PRKDC-/-^* cells as well as A549 cells pre-treated with 1 µM NU7441 were infected with ZIKV (m.o.i. 1) at 24 hours. As a positive control, cells were treated with 3 µM etoposide, a topoisomerase II inhibitor, for 12 hours (**Figures 2A, B**). As expected, etoposide treatment increased the number of γH2A.X foci (green) per cell, being enhanced in A549 *^PRKDC-/-^* or in NU7441-treated cells. However, the γH2A.X foci levels were similar in A549 cells infected with ZIKV (red) compared with uninfected cells. These results suggest that DNA-PKcs controls the ZIKV infection independent of any DSB-induced response.

### DNA-PKcs is required for IFN-I and IFN-III genes transcription during ZIKV infection

**Figure 3:**
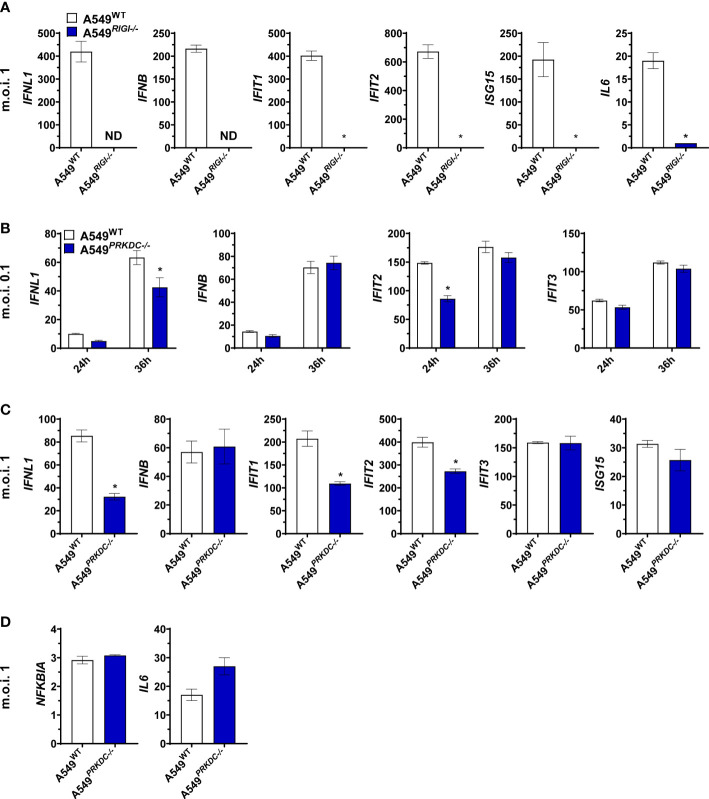
DNA-PKcs regulates interferon-related genes during ZIKV infection. **(A)** RT-qPCR to measure the expression of mRNA for indicated genes on A549^WT^ and A549^RIGI-/-^ cells infected with ZIKV m.o.i. 1 for 24 hours. **(B)** RT-qPCR to measure the expression of mRNA for indicated genes on A549^WT^ and A549*^PRKDC-/-^* cells infected with ZIKV m.o.i. 0.1 at the indicated time or **(C)** m.o.i. 1 at 24 hours. **(D)** Expression of mRNA for *NFKBIA* and *IL6*, determined by RT-qPCR on A549^WT^ and A549*^PRKDC-/-^* cells infected with ZIKV m.o.i. 1 at 24 hours. We used unpaired two-tailed Student’s t-test in **(A, C, D)**, and two-way ANOVA with Sidak’s correction in **(B)**. ND: Non-detected. * p<0.05, n = 3, error bars ± SEM.

RIG-I is the major ZIKV RNA sensor, leading to induction of IFN-I and IFN-III transcription, crucial for an efficient antiviral immune response (33, 34). To evaluate whether RIG-I orchestrates ZIKV sensing in A549 cells, we infected A549^WT^ and A549*^RIGI-/-^* with m.o.i. 1 for 24 hours to measure the induction of IFN-related genes transcription (**Figure 3A**). We observed A549 lacking RIG-I failed to induce IFN-I, IFN-III, ISGs, and *IL6* transcriptions during ZIKV infection, confirming that RIG-I is necessary for activation of antiviral immune response. Next, we investigated whether DNA-PKcs is required for activating the IFNs pathways downstream RIG-I during ZIKV infection. We performed a gene expression analysis of IFN-I, IFN-III, and ISGs in A549^WT^ and A549 *^PRKDC-/-^* cells infected with ZIKV at an m.o.i. 0.1 (**Figure 3B**) and m.o.i. 1 (**Figure 3C**). During ZIKV infection we observed a reduction of *IFNL1*, *IFIT1*, and *IFIT2*, but not *IFNB*, *IFIT3*, and *ISG15* transcription in the absence of DNA-PKcs. Similarly, in RPE cells, where ZIKV infection does not induce *IFNL1* transcription, the absence of DNA-PKcs decreased *IFNB* and *IFIT2*, but not *ISG15* transcription (**Figure S3A**). In addition, proinflammatory gene transcription induced after RIG-I activation such as *IL6* and *NFKBIA* is independent of DNA-PKcs in A549 cells upon ZIKV infection (**Figure 3D**), but dependent in RPE cells lacking DNA-PKcs (**Figure S3B**). Altogether, these results suggest crosstalk between RIG-I and DNA-PKcs during ZIKV infection, which results in an efficient antiviral immune response in A549 and RPE cells.

### IRF1, IRF3, and p65 nuclear accumulation during ZIKV infection is DNA-PKcs independent

**Figure 4:**
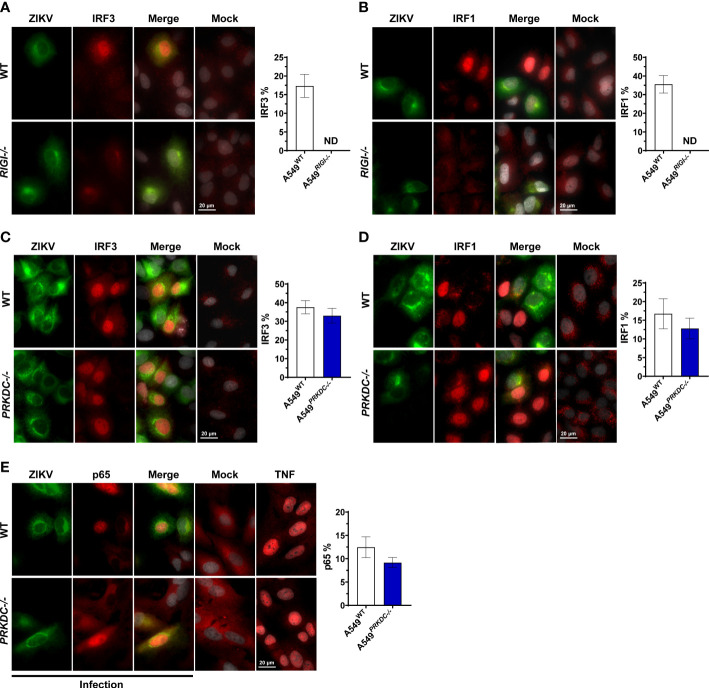
ZIKV induces IRF1, IRF3, and p65 nuclei accumulation independent of DNA-PKcs. Immunofluorescence analysis for localization of endogenous **(A)** IRF3 (red) and **(B)** IRF1 (red) on A549^WT^ and A549^RIGI-/-^ cells infected with ZIKV m.o.i. 1 (green, ZIKV-E protein) at 36 hours (left panel), and quantified by scoring cells with nuclear staining (right panel, n = 3, counts of at least 30 nuclei per slide). **(C–E)** Immunofluorescence analysis for localization of endogenous **(C)** IRF3 (red), **(D)** IRF1 (red), and **(E)** p65 (red) on A549^WT^ and A549*^PRKDC-/-^* cells infected with ZIKV m.o.i. 1 (green, ZIKV-E protein) at 24 hours (left panel), and quantified by scoring cells with nuclear staining (right panel, n = 3, counts of at least 30 nuclei per slide). TNF was used as a positive control in **(E)**. Cell nuclei were stained with DAPI (grey). We used unpaired two-tailed Student’s t-test. N = 3, error bars ± SEM. ND: non-detected.

We evaluated the crosstalk between RIG-I and DNA-PKcs during ZIKV infection analyzing the IFN-I/III-inducing transcription factors including IRF1, IRF3, IRF5, IRF7, and p65 (NF-κB subunit) (24, 25, 27, 29–31, 35). We infected A549 cells with ZIKV and measured the accumulation of the transcription factors in the nucleus. We observed that ZIKV infection induces IRF1 and IRF3, but not IRF5 and IRF7 nuclear accumulation (**Figures 4A–D** and **Figures S4A, B**). As expected, A549*^RIGI-/-^* cells failed to induce IRF3 and IRF1 nuclear accumulation, confirming that RIG-I is the major ZIKV intracellular sensor (**Figures 4A, B**). However, the activation of the transcription factors was independent of DNA-PKcs, showing a similar nuclear location percentage of IRF3 and IRF1 (**Figure 4C, D**). Similarly, the induction of p65 nuclei accumulation was independent of DNA-PKcs (**Figure 4E**). Interestingly, while IRF3 nuclei translocation occurred exclusively in infected cells, we observed that IRF1 accumulation in the nuclei occurred mostly in bystander cells, confirmed by ZIKV dsRNA staining, which shows the early stage of virus infection (**Figure S4C**). Altogether, these results suggest that upon ZIKV infection, DNA-PKcs is necessary for enhancement of IFNs and ISGs transcription, acting downstream activation of transcription factors.

## Discussion

The DNA-PK complex senses viral DNA with an unknown impact on ZIKV infection. Here we demonstrate that DNA-PKcs, the catalytic subunit of the DNA-PK complex, is critical for control of ZIKV infection by a non-canonical mechanism.

We analyzed different infection profiles such as quantifying viable ZIKV, intracellular ZIKV-RNA, ZIKV spreading to adjacent cells, and ZIKV-infected cells. We showed that absence of DNA-PKcs increases susceptibility to ZIKV infection in two different human epithelial cell lineages, allowing a faster virus spreading. Moreover, we showed that the DNA-PKcs mechanism for controlling ZIKV infection depends on its kinase function. Previous studies have associated DNA-PK complex with infection of flaviviruses. For instance, Vetter et al. (37) demonstrated that Ku70 and Ku80 knockdown in Huh7 cells are insufficient to impact DENV infection, differing from our findings with ZIKV (37). The difference could be explained due to a partial knockdown of the Ku components or the use of different cell lines and flavivirus. In addition, depletion of each DNA-PK subunit separately varies on number of differentially expressed genes (45). Hence, instead of Ku proteins, only the catalytic subunit may be critical for control of infection.

ZIKV infection induces DSB in human neural stem cells (8, 9), which may implicate DNA-PK activation (46). However, our findings showed that ZIKV does not increase H2A.X phosphorylated foci, a DSB marker, in human epithelial (A549) cells. Hence, ZIKV does not induce DSB in A549 cells, and the role of DNA-PKcs in controlling viral infection is independent of DSB repair response. In addition, we showed that either inhibition of DNA-PKcs kinase function or loss of whole protein has increased DSB sensitivity to etoposide, a genotoxic stressor, as suggested in previous studies (43, 47).

We showed that DNA-PKcs is necessary for an effective antiviral response against ZIKV infection which may explain its role in restricting the infection. The effect of DNA-PKcs on IFN-I and IFN-III transcriptions differs between cell types. Similar to our findings, Vetter et al. (37) demonstrated that inhibition of Ku80 protein expression decreases *IFNB* transcription in DENV-infected cells (37). It is important to notice that DNA-PKcs antiviral response might differ between humans and mice and virus species (19). These could explain why the antiviral response against other RNA viruses is DNA-PKcs independent (14). ZIKV infection might induce mitochondrial DNA release, activating DNA sensors such as DNA-PK or cGAS, which results in IFN-I and IFN-III transcription (14, 48–50). However, we observed abrogation of the IFNs transcription in A549*^RIGI-/-^* cells infected with ZIKV, suggesting only RNA sensing pathway is activated during the infection.

Our data show that ZIKV infection induces nuclear accumulation of the main transcription factors involved in IFN-I and IFN-III transcription independently of DNA-PKcs. These findings implicate that DNA or RNA sensing pathways upstream of the transcription factors are not affected by DNA-PKcs during ZIKV infection. A prior study suggested DNA-PK complex is not required for IRF3 nor p65 nuclear translocation under RNA virus infection or Poly(I:C) stimulation (14). Furthermore, we showed ZIKV infection fails to induce IRF1 and IRF3 accumulation to the nucleus in absence of the RNA sensor RIG-I, suggesting DNA-PKcs is not a ZIKV sensor receptor. Our findings confirmed that RIG-I is the major nucleic acid sensor activated by ZIKV, as previously demonstrated (33, 34), and suggests novel crosstalk between DNA-PKcs and RIG-I pathway downstream to transcription factors. One possibility is during ZIKV infection, DNA-PKcs acts in the nucleus regulating IFN transcription. The DNA-PK is known to be a regulator of the transcriptome and RNA metabolism (13, 38, 51–55). DNA-PKcs is described to phosphorylate RNA polymerase II, which might enhance the transcription of some viral genomes such as *Hepatitis B virus* and *Human immunodeficiency virus* (52, 55–57). Genotoxic stress or viral infections such as DENV induce DNA-PKcs localization to the nucleolus, where it acts as a regulator of pre-mRNA splicing (13, 37, 38). The regulation of RNA metabolism by DNA-PKcs should be explored in the future in the context of ZIKV infections.

Overall, these findings provide a role of DNA-PKcs in control of ZIKV virus infection beyond its DNA sensing function or DSB repair response. Future work should consider whether this conclusion can be generalized to different RNA viruses. This study will advance our understanding of the antiviral immune response and may contribute to new therapeutic approaches.

## Data availability statement

The original contributions presented in the study are included in the article/**Supplementary Materials**. Further inquiries can be directed to the corresponding author.

## Author contributions

DP, AZ-F, AB, BF and DM designed experiments and analyzed data. DP, GD, LG, DB, and DM performed experiments. GD, AZ-F, BT, HT and BF contributed with critical reagents/tools. DP and DM wrote the manuscript. All authors contributed to the article and approved the submitted version.

## Funding

Conselho Nacional de Desenvolvimento Científico e Tecnológico PhD studentship 141906/2017-0 to DP; Wellcome Trust PhD Studentship 203778/Z/16/Z to HT and BF, a UKRI/BBSRC research project grant BB/S001336/1 to BF.

## Acknowledgments

We thank Laboratório Multiusuário de Estudos em Biologia (LAMEB) for technical assistance. We thank the laboratory of immunobiology (LIM) members for their helpful discussions.

## Conflict of interest

The authors declare that the research was conducted in the absence of any commercial or financial relationships that could be construed as a potential conflict of interest.

## Publisher’s note

All claims expressed in this article are solely those of the authors and do not necessarily represent those of their affiliated organizations, or those of the publisher, the editors and the reviewers. Any product that may be evaluated in this article, or claim that may be made by its manufacturer, is not guaranteed or endorsed by the publisher.
