# Supplementary material for: DNA-PKcs restricts Zika virus spreading and is required for effective antiviral response
Source: Front Immunol. 2022 Oct 13;13:1042463. doi: 10.3389/fimmu.2022.1042463 (PMC9606669; doi:10.3389/fimmu.2022.1042463)
Supplement: Supplementary file 1 [file DataSheet_1.pdf]

1 Supplementary Table

Supplementary Table I. Primary antibodies used for immunofluorescence.

Antibody	Company	Code	Dilution
DNA-PKcs	Abcam	ab70250	1:200
IRF1	Abcam	ab186384	1:200
IRF3	Cell Signaling	#4302	1:200
IRF5	Abcam	ab181553	1:200
IRF7	Abcam	ab109255	1:200
p65	Santa Cruz	sc-8008	1:100
Phospho-Histone H2A.X (Ser139/Tyr142)	Cell Signaling	#5438	1:200
Anti-ZIKV-E (4G2)	Fiocruz-PR, Brazil	-	1:100
Human monoclonal antibody DV 18.4	Beltramello et al. 2010	-	1:100
dsRNA J2	Scicons	10010500	1:100

Supplementary Table II. Secondary antibodies used for immunofluorescence.

Antibody	Company	Code	Dilution
Goat anti-Rabbit IgG (H+L) Alexa Fluor 568 conjugated	Invitrogen	A-11011	1:2000
Goat anti-Mouse IgG (H+L) Alexa Fluor 488 conjugated	Invitrogen	A-11001	1:2000
Rabbit anti-Mouse IgG (H+L) Alexa Fluor 568 conjugated	Invitrogen	A-11061	1:2000
Goat anti-Human IgG (H+L) Alexa Fluor 488 conjugated	Invitrogen	A-11013	1:2000

Supplementary Table III. qPCR primer sequences for *homo sapiens*.

Gene symbol	Gene name	Primer Direction	Primer sequence
GAPDH	glyceraldehyde-3-phosphate dehydrogenase	Forward	TCGGAGTCAACGGATTTGGT
		Reverse	TGAAGGGGTCATTGTATGGCA
IFNB	interferon beta 1	Forward	AAACTCATGAGCAGTCTGCA
		Reverse	AGGAGATCTTCAGTTTCGGAGG
IFNL1	interferon lambda 1	Forward	TTCCAAGCCCACCACAACTG
		Reverse	GAGTGACTCTTCCAAGGCGT
NFKBIA	NFKB inhibitor alpha	Forward	TACACCTTGCCTGTGAGCAG
		Reverse	TAGCCTTCAGGATCAGGATGGAG TGG
IFIT1	interferon-induced protein with tetratricopeptide repeats 1	Forward	GCAGCCAAGTTTTACCGAAG
		Reverse	AGCCCTATCTGGTGATGCAG
IFIT2	interferon induced protein with tetratricopeptide repeats 2	Forward	GACACGGTTAAAGTGTGGAGG
		Reverse	TCCAGACGGTAGCTTGCTATT
IFIT3	interferon induced protein with tetratricopeptide repeats 3	Forward	AGAAAAGGTGACCTAGACAAAGC
		Reverse	CCTTGTAGCAGCACCCAATCT
ISG15	ISG15 ubiquitin like modifier	Forward	AATGCGACGAACCTCTGAAC
		Reverse	GAAGGTCAGCCAGAACAGGT
IL6	interleukin 6	Forward	CCACACAGACAGCCACTCAC
		Reverse	AGGTTGTTTTCTGCCAGTGC
ZIKV	zika virus	Forward	CTGTGGCATGAACCCAATAG
		Reverse	ATCCCATAGAGCACCACTCC

Supplementary Table IV. Primary antibodies used for immunoblotting.

Antibody	Company	Code	Dilution
DNA-PKcs	Abcam	ab70250	1:500
Tubulin	Millipore	05-829	1:5000

Supplementary Table V. Secondary antibodies used for immunoblotting.

Antibody	Company	Code	Dilution
HRP-linked anti-rabbit	Cell Signaling	7074	1:5000
HRP-linked anti-mouse	Cell Signaling	7076	1:5000
