# Supplementary material for: DNA-PKcs restricts Zika virus spreading and is required for effective antiviral response
Source: Front Immunol. 2022 Oct 13;13:1042463. doi: 10.3389/fimmu.2022.1042463 (PMC9606669; doi:10.3389/fimmu.2022.1042463)
Supplement: Supplementary file 2 [file DataSheet_2.pdf]

1 Supplementary Figures

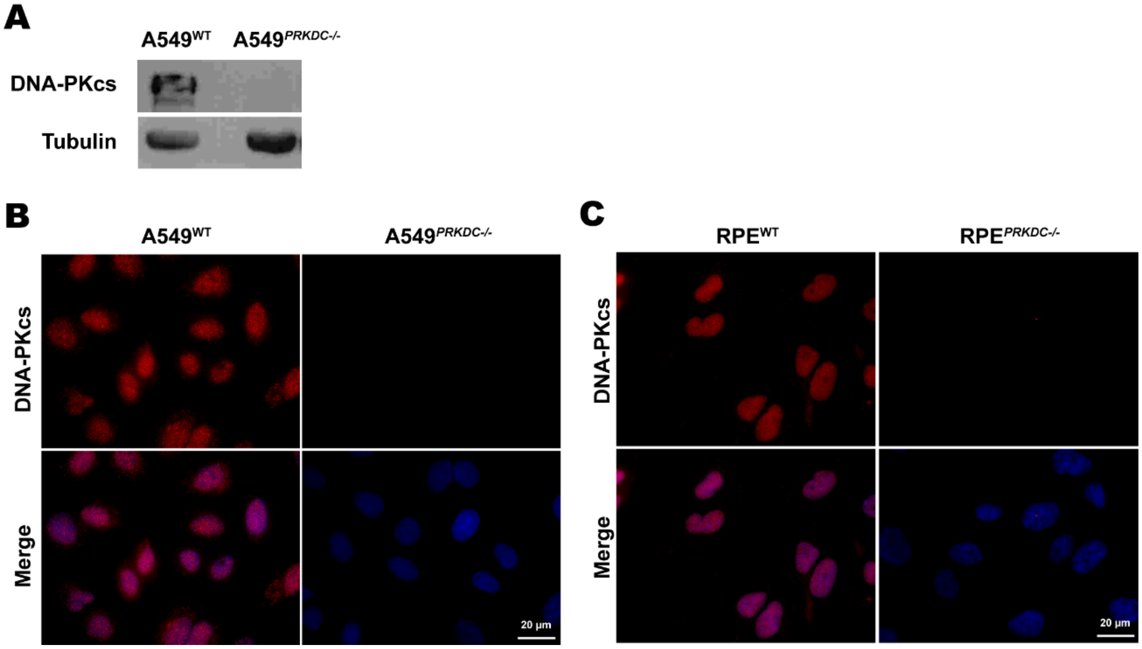

Supplementary Figure 1. DNA-PKcs deficient cell generation. (A) DNA-PKcs expression in whole-cell extracted proteins from A549^{WT} and A549$^{PRKDC-/-}$ cells were analyzed by immunoblotting. Immunofluorescence analysis of DNA-PKcs presence (red) on **(B)** A549^{WT} and A549$^{PRKDC-/-}$ or **(C)** RPE^{WT} and RPE$^{PRKDC-/-}$ cells. Cell nuclei were stained with DAPI (blue).

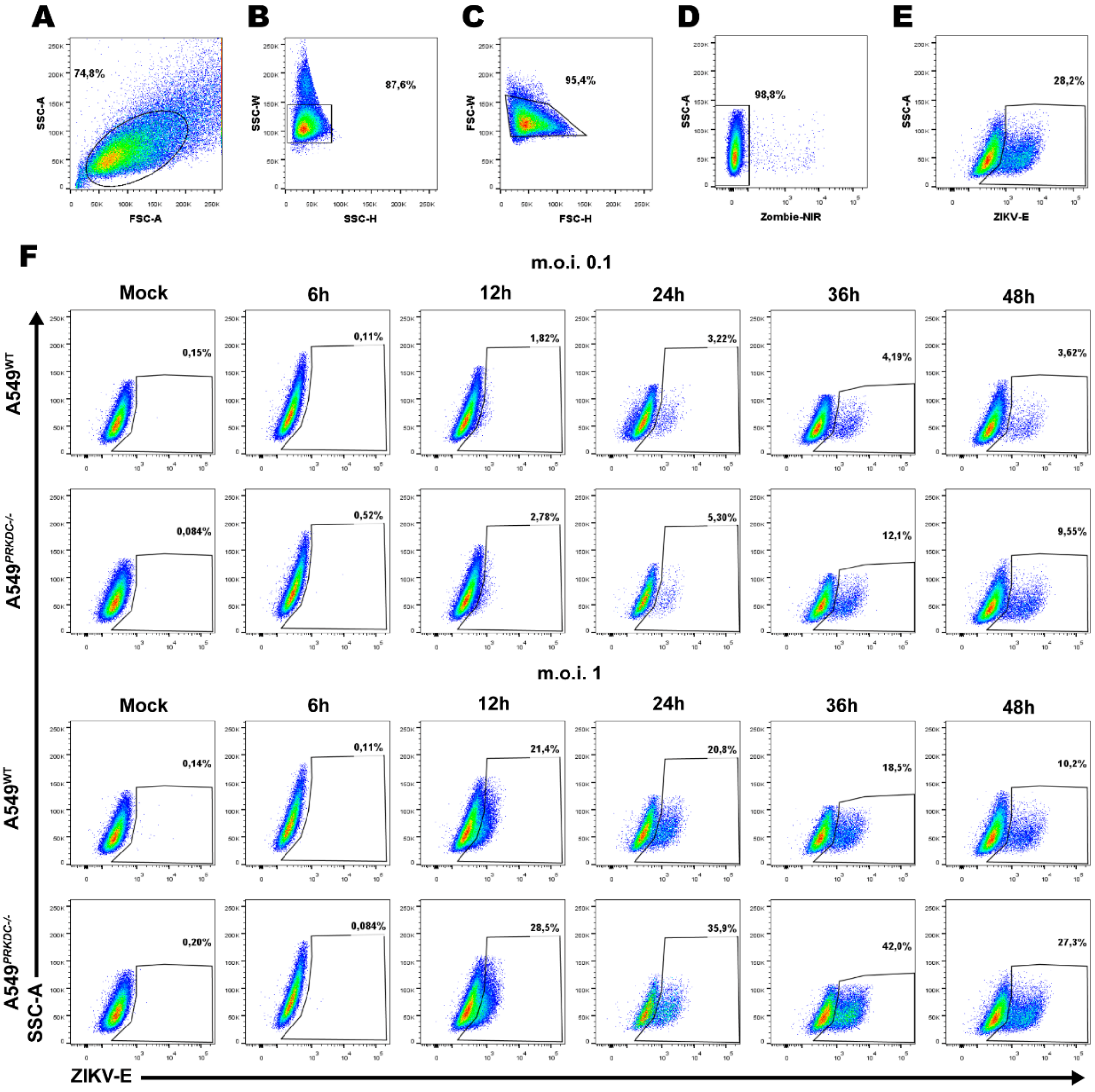

Supplementary Figure 2. Flow cytometry analysis. Gating strategy, which **(A)** cell population selection, **(B, C)** doublet exclusion, **(D)** Exclusion of non-viable cells stained with Zombie^{NIR}, and **(E)** selecting ZIKV-infected cells staining with ZIKV-E primary antibody and AlexaFluor488 secondary antibody. **(F)** A549^{WT} and A549$^{PRKDC-/-}$ cells infected with ZIKV at indicated m.o.i. and time. Representative of three replicates.

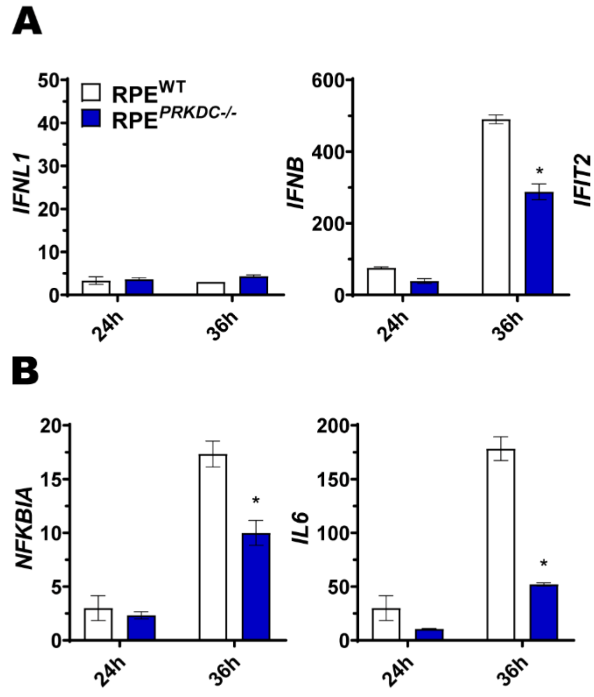

Supplementary Figure 3. DNA-PKcs regulates interferon-related genes during ZIKV infection in RPE cells. RT-qPCR to measure the expression of mRNA for indicated genes on RPE^{WT} and $RPE^{PRKDC-/-}$ cells infected with ZIKV **(A)** m.o.i. 1 at the indicated time. **(B)** Expression of mRNA for *NFKBIA* and *IL6*, determined by RT-qPCR on RPE^{WT} and $RPE^{PRKDC-/-}$ cells infected with ZIKV in m.o.i. 1 at indicated time. We used two-way ANOVA with Sidak's correction. * $p<0.05$, n = 3, error bars ± SEM.

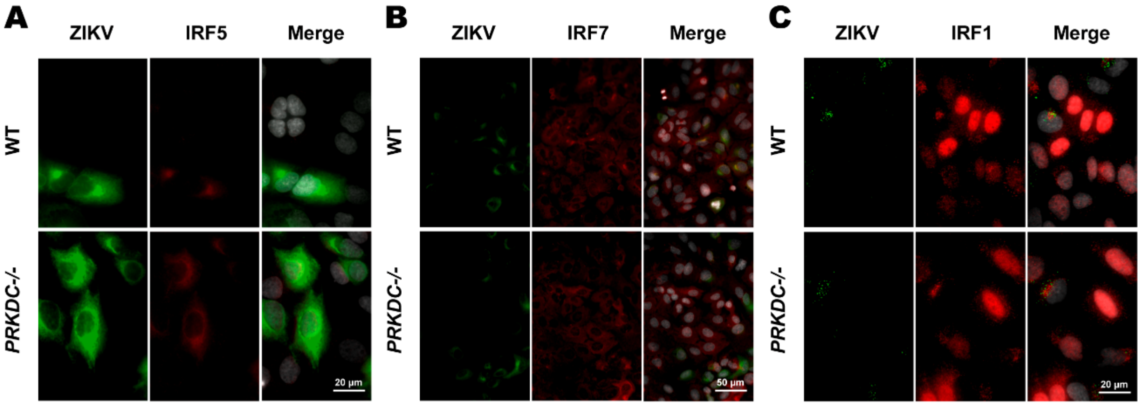

Supplementary Figure 4. ZIKV infection fails to induce IRF5 and IRF7 nuclei accumulation, and ZIKV-induced IRF1 nuclei accumulation on bystander cell independent of DNA-PKcs. Immunofluorescence analysis for localization of endogenous **(A)** IRF5 (red), and **(B)** IRF7 (red) on $A549^{WT}$ and $A549^{PRKDC-/-}$ cells infected with ZIKV (green, ZIKV-E protein) in m.o.i. 1 at 24 hours. **(C)** Immunofluorescence analysis for localization of endogenous IRF1 on $A549^{WT}$, $A549^{PRKDC-/-}$ cells infected with ZIKV (green, dsRNA) m.o.i. 1 at 6 hours. Cell nuclei were stained with DAPI (grey).
